# A *de novo* reference assembly of the yellow mangrove *Ceriops zippeliana* genome

**DOI:** 10.1093/g3journal/jkac025

**Published:** 2022-02-02

**Authors:** Wirulda Pootakham, Chutima Sonthirod, Chaiwat Naktang, Wasitthee Kongkachana, Sonicha U-thoomporn, Phakamas Phetchawang, Chatree Maknual, Darunee Jiumjamrassil, Tamanai Pravinvongvuthi, Sithichoke Tangphatsornruang

**Affiliations:** 1 National Omics Center, National Science and Technology Development Agency (NSTDA), Pathum Thani 12120, Thailand; 2 Department of Marine and Coastal Resources, Bangkok 10210, Thailand

**Keywords:** *Ceriops zippeliana*, mangrove, chromosome-scale genome assembly, 10x Genomics, RagTag

## Abstract

Mangroves are of great ecological and economical importance, providing shelters for a wide range of species and nursery habitats for commercially important marine species. *Ceriops zippeliana* (yellow mangrove) belongs to Rhizophoraceae family and is commonly distributed in the tropical and subtropical coastal communities. In this study, we present a high-quality assembly of the *C. zippeliana* genome. We constructed an initial draft assembly of 240,139,412 bases with an N50 contig length of 564,761 bases using the 10x Genomics linked-read technology. This assembly was further scaffolded with RagTag using a chromosome-scale assembly of a closely related *Ceriops* species as a reference. The final assembly contained 243,228,612 bases with an N50 scaffold length of 10,559,178 Mb. The size of the final assembly was close to those estimated using DNA flow cytometry (248 Mb) and the *k-*mer distribution analysis (246 Mb). We predicted a total of 23,474 gene models and 21,724 protein-coding genes in the *C. zippeliana* genome, of which 16,002 were assigned gene ontology terms. We recovered 97.1% of the highly conserved orthologs based on the Benchmarking Universal Single-Copy Orthologs analysis. The phylogenetic analysis based on single-copy orthologous genes illustrated that *C. zippeliana* and *Ceriops tagal* diverged approximately 10.2 million years ago (MYA), and their last common ancestor and *Kandelia obovata* diverged approximately 29.9 MYA. The high-quality assembly of *C. zippeliana* presented in this work provides a useful genomic resource for studying mangroves’ unique adaptations to stressful intertidal habitats and for developing sustainable mangrove forest restoration and conservation programs.

## Introduction

Mangrove forests are the most dominant intertidal ecosystems distributed in the regions between land and sea in the tropical and subtropical zones (Giri *et al.* 2011). They are one of the most biologically productive and complex ecosystems, and their roles in protecting against natural disasters such as hurricanes and tsunami are well recognized (Giri 2021). Mangrove forests are continuously affected by human exploitation and deforestation as well as catastrophic climate changes such as sea level elevation and increase in seawater temperature (Krauss *et al.* 2008). The overexploitation of mangrove resources emphasizes the need for effective conservation and restoration management systems. Successful and sustainable managements of mangrove forests require biological and molecular knowledge of the species that are members of mangrove ecosystems. The development of genomic resources for mangroves provides a strong foundation for future studies on the population structures and comparative genomic analyses. Several research groups have recently sequenced mangrove genomes from multiple lineages, including *Rhizophora apiculata* (Xu *et al.* 2017), *Kandelia obovata* (Hu *et al.* 2020), *Avicennia marina* (Friis *et al.* 2021), *Aegiceras corniculatum* (Feng *et al.* 2021), and *Bruguiera parviflora* (Pootakham *et al.* 2022). There have also been reports on transcriptomic resources from various mangrove species in the past few years (Dassanayake *et al.* 2009; Yamanaka *et al.* 2009; Yang *et al.* 2015a, 2015b; Krishnamurthy *et al.* 2017; Xu *et al.* 2017). *Ceriops zippeliana* (yellow mangrove) is a mangrove tree belonging to the family Rhizophoraceae, the most mangrove-rich taxon. *Ceriops* *zippeliana* is widely distributed in the Indo-West regions. Limited availability of genetic and genomic information on *C. zippeliana* renders the studies on molecular mechanisms underlying its adaption to harsh environment and the investigation of its genetic diversity and population structure rather difficult. Here, we utilized the 10x Genomics technology to obtain an initial draft assembly of the *C. zippeliana* genome, which was subsequently scaffolded to a chromosome-level assembly using a Hi-C genome assembly of *Ceriops tagal* as a reference. The final assembly of the *C. zippeliana* genome contained 243,228,612 bases with an N50 scaffold length of 10,559,178 Mb. The availability of this high-quality genome assembly provides a valuable resource for phylogenetic and genetic diversity studies.

## Materials and methods

### Plant material collection and extraction of nucleic acid

Leaf tissues from a mature *C. zippeliana* (12°31′33.6″N 102°05′45.8″E) in the International Mangrove Botanical Garden Rama IX (Chantaburi, Thailand) was collected, flash-frozen and stored in liquid nitrogen until use. High molecular weight genomic DNA were extracted using the QIAGEN Genomic-tip 100/G following the manufacterer’s protocol (Qiagen, Hilden, Germany). The integrity of the DNA samples was evaluated with the Pippin Pulse Electrophoresis System (Sage Science, Beverly, MA, USA) prior to the 10x Genomics library preparation.

For transcriptome sequencing, we isolated total RNA from leaf tissues collected from the same individuals used for genome sequencing following the protocol in Pootakham *et al.* (2021b). Briefly, total RNA was isolated using the CTAB buffer (2% CTAB, 1.4M NaCl, 2% PVP, 20 mM EDTA pH 8.0, 100 mM Tris-HCl pH 8.0, 0.4% SDS). RNA was extracted from the aqueous phase 3 times using 25:24:1 phenol: chloroform: isoamylalcohol and precipitated overnight in ¼ volumes of 8M LiCl. The pellets were washed with 70% ethanol, air-dried and resuspended in RNase-free water. Poly(A) mRNA was enriched using the Dynabeads mRNA Purification Kit (ThermoFisher Scientific, Waltham, MA, USA). Prior to the MGISEQ library construction, the integrity of the RNA samples was assessed on the Fragment Analyzer System (Agilent, Santa Clara, CA, USA).

### Library preparation and sequencing

For whole-genome sequencing, 1.25 ng of high integrity, high molecular weight DNA was used for the 10x Genomics linked-read library preparation using the Chromium Genome Library Kit & Gel Bead Kit v2, the Chromium Genome Chip Kit v2 and the Chromium i7 Multiplex Kit according to the manufacturer’s instructions (10x Genomics, Pleasanton, CA, USA). The 10x Genomics library was sequenced on the Illumina HiSeq X Ten (150 bp paired-end reads). To obtain short-read RNA sequences, the sequencing library was prepared according to the method reported in Pootakham *et al.* (2021b). A total of 200 ng of poly(A) mRNA was used to construct a library using the MGIEasy RNA Library Prep Kit V3.0 (MGI Tech, Shenzhen, China). The library was subsequently sequenced using the MGISEQ-2000RS Sequencing Flow Cell V3.0 and the MGISEQ-2000RS (MGI Tech, Shenzhen, China).

### Preliminary genome assembly and scaffolding

Linked-read Illumina sequence data were assembled using the Supernova assembler version 2.1.1 using the default settings (https://support.10xgenomics.com/de-novo-assembly/software/pipelines/latest/using/running; last accessed: February 7, 2022; 10x Genomics, Pleasanton, USA). The 10x Genomics assembly was further scaffolded with RagTag version 1.1.0 (https://github.com/malonge/RagTag; last accessed: February 7, 2022) (Alonge *et al.* 2019) using a chromosome-level assembly of *C. tagal* as a reference.

### Genome size estimation

Two methods were used to estimate the nuclear genome sizes in *C. zippeliana*: the DNA flow cytometry and the *k*-mer analysis of the sequencing read distribution. For DNA flow cytometry analysis, fresh leaf tissues were chopped into small pieces with a sharp razor blade to release nuclei, and the procedure in Dolezel and Bartos (2005) was followed. The woody plant buffer (WPB) reported in Loureiro *et al.* (2007) was used as a nuclear isolation buffer for *C. zippeliana*. The nuclei were stained with 50 μg/mL propidium iodide (Thermo Fisher Scientific, Waltham, MA, USA). The LB01 buffer was used as the nuclear isolation buffer for Arabidopsis (the DNA reference standard) (Dolezel and Bartos 2005). The *k*-mer analysis was performed using the Jellyfish software version 2.2.10, and the *k-*mer distribution was plotted with GenomeScope version 1.0 (*k *=* *19; http://qb.cshl.edu/genomescope/; last accessed: February 7, 2022) (Vurture *et al.* 2017).

### Evaluation of the genome assembly quality

We evaluated the quality of the final assembly by aligning short-read DNA sequences (Illumina data) and Trinity-assembled transcript sequences using BWA version 0.7.17-r1188 and GMAP version 2020-09-12 (Wu and Watanabe 2005), respectively. We also used the Benchmarking Universal Single-Copy Orthologs (BUSCO version 4.0.5) (Simão *et al.* 2015) to evaluate the assembly by testing for the presence and completeness of the orthologs using the plant-specific database from the Embryophyta OrthoDB release 10 (Kriventseva *et al.* 2015).

### Repetitive sequence identification

RepeatModeler version 2.0.2 (http://www.repeatmasker.org/RepeatModeler/; last accessed: February 7, 2022) was used to construct a *de novo* repeat library and identify transposable element (TE) families in the unannotated genome assembly. RECON version 1.08 and RepeatScout version 1.0.5 were used to identify the boundaries of repetitive elements and to build consensus models of interspersed repeats. We aligned the repeat sequences in the library to GenBank’s nr protein database using BLASTX with an *e*-value cutoff of 10^−6^ to confirm that did not contain large families of protein-coding sequences that were not TEs.

### Gene prediction and annotation

Following the annotation protocol described in Pootakham *et al.* (2021a), we used evidences from homology-based prediction, RNA-based prediction, and ab initio prediction to identify protein-coding sequences in the unmasked genome assembly using EvidenceModeler (EVM) version 1.1.1 (Haas *et al.* 2008). RNA-based prediction approaches utilized RNA-seq data from *C. zippeliana*. Short-read RNA sequences were mapped to the assembly during the initial step of annotation using the PASA2 pipeline version 2.4.1 (Haas *et al.* 2008). Protein sequences from *Populus trichocarpa*, *Sesamum indicum*, *Mimulus guttatus*, *Eucalyptus grandis*, and *Oryza sativa* obtained from public databases were aligned to the unmasked genome using AAT (Huang *et al.* 1997). Two *ab initio* gene predictors were run on the unmasked assembly. Protein-coding gene predictions were obtained with Augustus version 3.3.3 (Stanke *et al.* 2004) trained with *P. trichocarpa, S. indicum*, *M. guttatus*, *E. grandis*, *O. sativa*, and PASA (version 2.4.1) transcriptome alignment assembly (Hoff *et al.* 2016, 2019) using RNA-seq alignment files as inputs. All gene predictions were combined by EVM to generate consensus gene models using the following weights for each evidence type: PASA2–1, AAT—0.3, and Augustus—0.3. We cross-examined the position of annotated genes with those of known repeats and excluded any gene that had more than 50% overlapping sequences with repetitive elements from the list. The remaining predicted genes were functionally annotated using OmicsBox version 2.0.10 (https://www.biobam.com/download-omicsbox/; last accessed: February 7, 2022). Protein sequences were aligned with UniProtKB/Swiss-Prot (swissprot v5) and GenBank nonredundant database (nr v5) using local BLASTP with an e-value cutoff of 10^−5^. Gene ontology (GO) terms were retrieved and assigned to *C. zippeliana* query sequences, and enzyme codes (EC) corresponding to GO were retrieved and mapped to KEGG pathway annotations.

### Phylogenetic analysis

To perform the phylogenetic analysis, OrthoFinder was used to identify orthologous groups in *C. zippeliana*, five mangrove species [*Bruguiera gymnorhiza* (Yamanaka *et al.* 2009)*, B. parviflora* (Pootakham *et al.* 2022), *C. tagal* (Yang *et al.* 2015b), *K. obovata* (Hu *et al.* 2020)*, R. apiculata* (Xu *et al.* 2017)], five dicots (*Arabidopsis thaliana, Cucumis melo, Cucumis sativus*, *Ricinus communis, P. trichocarpa*) and one monocot (*O. sativa*). A phylogenetic tree was constructed based on protein sequences from single-copy orthologous groups using the RAxML-NG program (Stamatakis 2006). Protein sequences in each single-copy orthologous group were aligned with MUSCLE (Edgar 2004), and the alignment gaps were removed with trimAl (Capella-Gutiérrez *et al.* 2009) using the automated1 heuristic method. The catsequences program (https://github.com/ChrisCreevey/catsequences; last accessed: February 7, 2022) was used to concatenate the alignment blocks, and the substitution model for each block was estimated using the ModelTest-NG program (Darriba *et al.* 2020). The outputs were then used to compute a maximum-likelihood phylogenetic tree. Divergence times were estimated using the MCMCtree software version 4.0 [PAML 4 package (Yang 2007)] using the Bayesian Relaxed Molecular Clock (BRMC) approach. The “correlated molecular clock” and “JC69” models were used with the published divergence time between the common ancestor of Rhizophoraceae, Euphorbiaceae (*R. communis*), and Salicaceae (*P. trichocarpa*) estimated at 105–120 million years ago (MYA) (Davis *et al.* 2005; Xi *et al.* 2012). The most recent fossil recognized as ancestral *Rhizophora* has been dated to the late Eocene (33.9–38 MYA) (Muller 1981; Graham 2006). We used approximate likelihood calculations based on the gradient and Hessian matrix of the likelihood at the maximum likelihood estimates of branch lengths, calculated with CODEML (within the PAML package). Gaps and ambiguity characters will be treated as missing data in the likelihood calculation. Priors on the mean (or ancestral) rate “rgene_gamma” were set to G (2 20 1), corresponding to a diffuse prior with mean rates of 1 substitution per 100 million years. The tree prior assumed a uniform birth-death process with default parameters. Two independent MCMC chains were run for 2 million cycles, sampling every 10 iterations, after the initial 2,000 cycles that were discarded as burning.

Significantly expanded/contracted gene families across the phylogenetic tree (*P*-value < 0.05) were calculated using the CAFE software version 5 (Han *et al.* 2013) with the gene birth-death (λ) parameters estimated using the maximum-likelihood method. A phylogenetic tree with divergence time from the MCMCTree output was used as a probabilistic model to infer family expansion and contraction. To estimate relative timing of evolutionary divergence between *C. zippeliana* and closely related mangrove species, we used the 4DTv approach to analyze orthologous gene pairs. The transversion of 4-fold degenerate synonymous sites (4DTv) were calculated for each gene pair from the aligned blocks using the in-house perl script (Pootakham *et al.* 2021a). We defined collinear blocks as regions of the genome that contained at least 10 collinear genes with fewer than 6 intervening genes.

### The analysis of genome synteny

MCscanX (Wang *et al.* 2012) was used to analyze colinearity within the *C. zippeliana* genome. To identify putative paralogs, *C. zippeliana* amino acid sequences were aligned against themselves using BLASTP with an *e*-value cutoff of 10^−10^. We defined intragenic homeologous segments as regions of at least 10 genes with colinear or nearly colinear runs of paralogs elsewhere in the genome with fewer than 6 intervening genes. The intragenic homeologous segments were plotted using CIRCOS version 0.69.8 (Krzywinski *et al.* 2009).

## Results and discussion

### Genome assembly and quality assessment

**Fig. 1.**
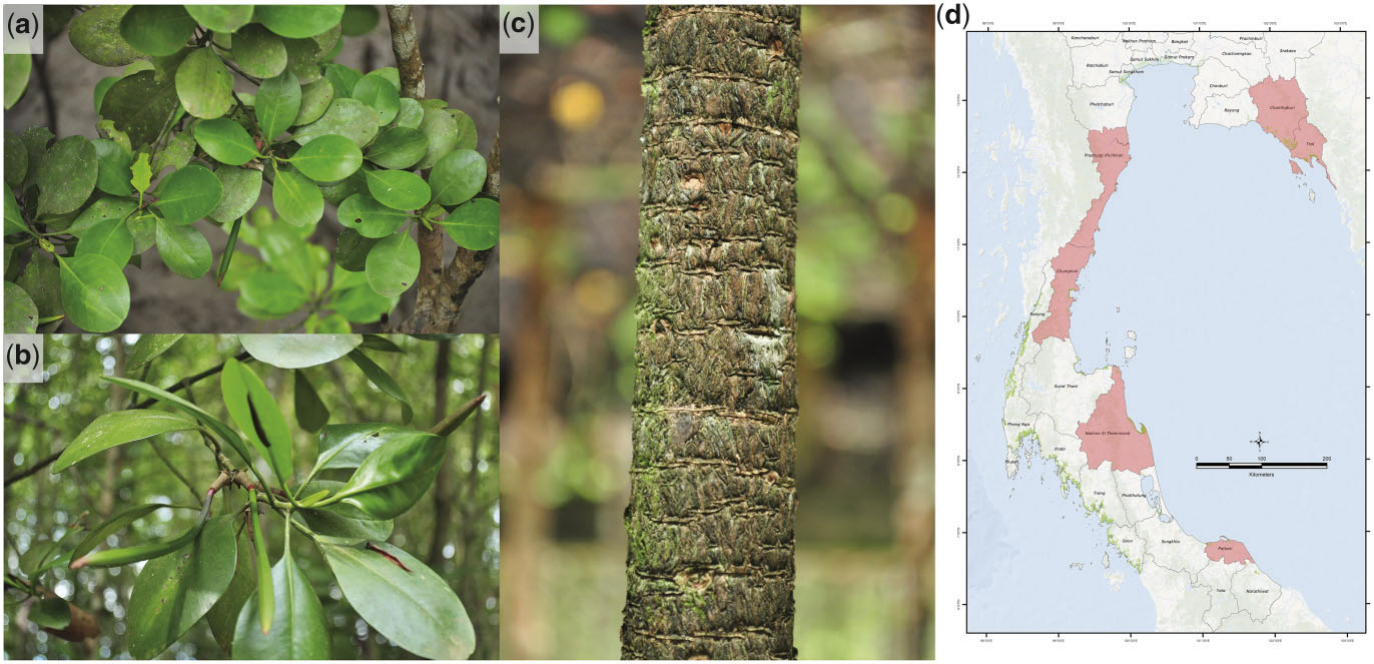
Morphology and distribution of *C. zippeliana*. a) Leaves, b) Fruits, c) Trunk, and d) Distribution map of *C. zippeliana* in Thailand.

**Fig. 2.**
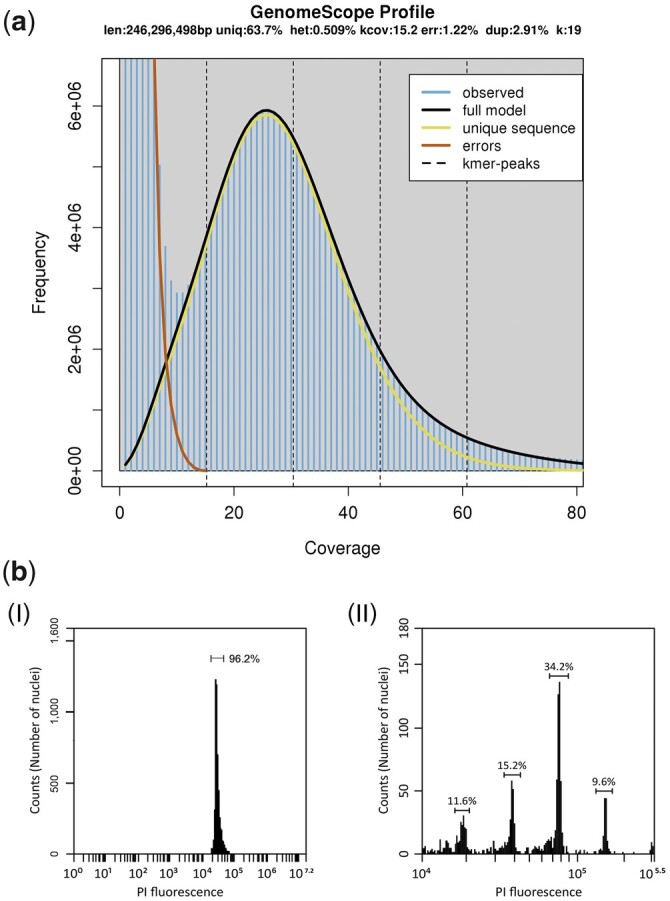
Genome size estimation. a) 19-mer estimate of the genome size. The *x*-axis is depth coverage (X), and the *y-*axis is the total number of *k*-mers with a given frequency. b) Histograms of relative DNA contents obtained after analysis of nuclei isolated from young leaf tissues of (I) *C. zippeliana* and (II) *A. thaliana* (used as a reference standard).

**Fig. 3.**
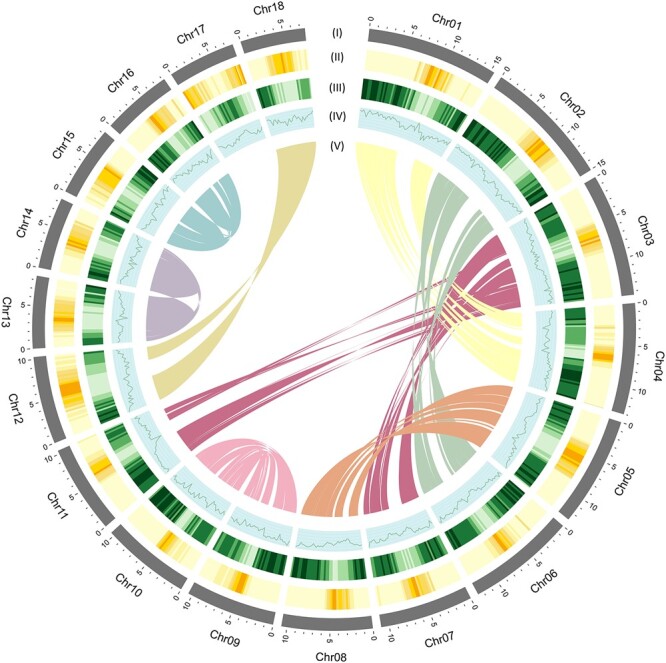
Genomic landscape of *C. zippeliana.* (I) A physical map of 18 chromosomes numbered according to size (Mb scale). (II) Repeat density represented by the fraction of genomic regions covered by repetitive sequences in 250-kb windows. (III) Gene density represented by the number of genes in 250-kb windows. (IV) GC content represented by the percentage of G + C bases in 250-kb windows. (V) Syntenic regions in the genome are illustrated by connected lines.

To generate an initial assembly of the *C. zippeliana* genome, high-quality genomic DNA from a single mature individual in the International Mangrove Botanical Garden Rama IX (Chantaburi, Thailand; Fig. 1) was used for 10x Genomics linked-read library preparation. A total of 305,048,562 paired-end reads totaling 89.68 Gb was obtained. The preliminary assembly generated from the linked-read data was 240,139,412 bases with N50 contig lengths of 564,761 bases (Table 1). The initial assembly was subsequently scaffolded with RagTag (Alonge *et al.* 2019) using a chromosome-level assembly of a closely related mangrove species, *C. tagal*, as a reference (Pootakham W, Naktang C, Sonthirod C, Kongkachana W, Narong N, Sangsrakru D, Maknual C, Jiumjamrassil D, Chumriang P, Tangphatsornruang S, manuscript under review). The *C. zippeliana* final assembly contained 243,228,612 bases in 25,704 scaffolds with an N50 length of 10,559,178 bases (Table 1). There were substantial improvements in assembly metrics in the final assembly. For instances, the N50 scaffold length increased 18.7-fold, and the length of the longest scaffold increased 6-fold in the final assembly. The 18 largest pseudomolecules, corresponding to haploid chromosome number (2*n *=* *36, *n *=* *18) in *Ceriops* species (Das *et al.* 1995; Basak *et al.* 1998), covered 195,638,481 or 80.43% of the 243-Mb *C. zippeliana* assembly. From here on, these pseudomolecules will be referred to as chromosomes, numbered according to size. We estimated the size of the *C. zippeliana* genome based on the *k*-mer frequency histogram derived from short-read Illumina sequences and DNA flow cytometry (Fig. 2). The assembly size (243 Mb; Fig. 3) corresponded well with both the DNA flow cytometry method (248 Mb) and the analysis of *k*-mer distribution of sequencing reads (246 Mb).

**Table 1.** *Ceriops zippeliana* genome assembly statistics.

	10x Genomics	10x Genomics + RagTag scaffolding
N50 contig/scaffold size (bases)	564,761	10,559,178
L50 contig/scaffold number	121	10
N75 contig/scaffold size (bases)	172,342	8,031,761
L75 contig/scaffold number	291	17
N90 contig/scaffold size (bases)	2,565	2,690
L90 contig/scaffold number	4,376	3,243
Assembly size (bases)	240,139,412	243,228,612
Number of scaffolds	27,067	25,704
Number of scaffolds ≥100 kb	358	29
Number of scaffolds ≥1 Mb	45	18
Number of scaffolds ≥10 Mb	0	12
Longest scaffold (bases)	2,579,863	15,634,258
%N	1.62	1.66
GC content (%)	35.24	35.24

The accuracy of the *C. zippeliana* assembly was assessed by mapping short-read DNA sequences and assembled transcripts to the genome. We observed that 95.08% and 86.11% of the Illumina short-read sequences and the Trinity-assembled transcripts, respectively, could be aligned to the genome assembly, suggesting that our genome assembly is of high quality. *C. zippeliana* gene predictions recovered 97.1% of the highly conserved orthologs based on the BUSCO analysis (93.9% classified as complete and single-copy, 1.7% as complete and duplicated, and 1.5% as fragmented) while 2.9% of the Embryophyta conserved orthologs were missing from the gene prediction.

### Repetitive DNA

The total length of repetitive sequences accounted for 39.23% of the *C. zippeliana* genome (94.22 Mb; Table 2). These repeat elements contained 0.97 Mb (0.41%) of DNA transposons, 47.91 Mb (19.94%) of retrotransposons, 4.16 Mb (1.73%) of simple sequence repeats, 41.18 (17.15%) of unclassified elements (Table 2). As reported for several other plant genomes, retrotransposons were the majority of the classified repetitive sequences, representing approximately half (50.85%) of the total repeat elements in the *C. zippeliana* genome. Most of the retrotransposons were classified as long terminal repeats (LTR; 41.03 Mb), which could further be categorized into *Copia*-like (26 Mb) and *Gypsy*-like (13.23 Mb) elements (Table 2). The percentage of repetitive elements in the *C. zippeliana* genome (39.23%) was considerably higher than that observed in the *R. apiculata* (29%) (Xu *et al.* 2017), *B. parviflora* (26%) (Pootakham *et al.* 2022) and *K. obovata* (24%) (Hu *et al.* 2020) genomes but similar to the number reported for the *P.* *trichocarpa* genome (40%) (Zhou and Xu 2009).

**Table 2.** Repeat contents in the *C. zippeliana* genome assembly.

Types of repeats	Bases (Mb)	% of the assembly	% of total repeats
DNA transposons	0.97	0.41	1.03
Retrotransposons			
LINE	6.74	2.81	7.15
SINE	0.14	0.06	0.15
LTR: *Copia*	26.00	10.82	27.60
LTR: *Gypsy*	13.23	5.50	14.04
LTR: Others	1.8	0.75	1.91
Simple sequence repeats	4.16	1.73	4.41
Unclassified elements	41.18	17.15	43.71
Total	94.22	39.23	—

### Genome annotation, gene prediction, and noncoding RNAs

We predicted a total of 23,474 gene models (Table 3) and annotated 21,724 protein-coding genes (92.54% of the predicted gene models; Table 4). The remaining models did not display homology to known genes and were considered hypothetical proteins. Gene ontology had been assigned to 16,002 protein-coding genes (68.17% of the predicted gene models). Of the predicted gene models, 72.33%, 29.62%, and 17.79% could be functionally annotated using the Swissprot, EC, and KEGG databases, respectively (Table 4). In addition, Infernal 1.1 (Nawrocki and Eddy 2013) was used to perform a homology search and annotate noncoding RNA sequences (Table 5). A total of 3,536 microRNAs, 310 ribosomal RNAs, 404 transfer RNAs and 12,566 small nuclear RNAs were identified in the genome. The average GC content of the *C. zippeliana* genome assembly was 35.24% (Table 1), which was close to the average GC content in introns (34.85%) while the average GC content in exons was higher at 45.35% (Table 3).

**Table 3.** Annotation statistics for *C. zippeliana*.

	*C. zippeliana*
Number of predicted gene models	23,474
Total gene length (Mb)	70.26
Average gene size (nt)	2,993
Average number of exons/gene	5.415
Total exon length (Mb)	29.33
Average exon length (nt)	230.8
GC content of exons (%)	45.35
Average number of Introns/gene	4.41
Total intron length (Mb)	40.95
Average intron length (nt)	395.2
GC content of introns (%)	34.85

**Table 4.** Functional annotations of *C. zippeliana* protein-coding genes.

Database	Number of genes annotated (% of all predicted genes)
NR	21,724 (92.54%)
Swissprot	16,978 (72.33 %)
GO	16,002 (68.17 %)
EC	6,953 (29.62%)
KEGG	4,177 (17.79%)
unannotated genes	1,750 (7.46%)
Total (predicted gene models)	23,474

**Table 5.** Noncoding RNAs in the *C. zippeliana* genome.

Type	Number	Length (nt)	Mean length (nt)
rRNA	310	93,420	301.35
microRNA	3,536	345,228	97.63
snRNA	12,566	789,329	62.81
snoRNA	12,349	767,418	62.14
splicing	217	21,911	100.97
tRNA	404	29,208	72.30
other ncRNA	5,331	435,191	81.63

### Comparative analysis of orthologous genes and phylogenetic relationships

**Fig. 4.**
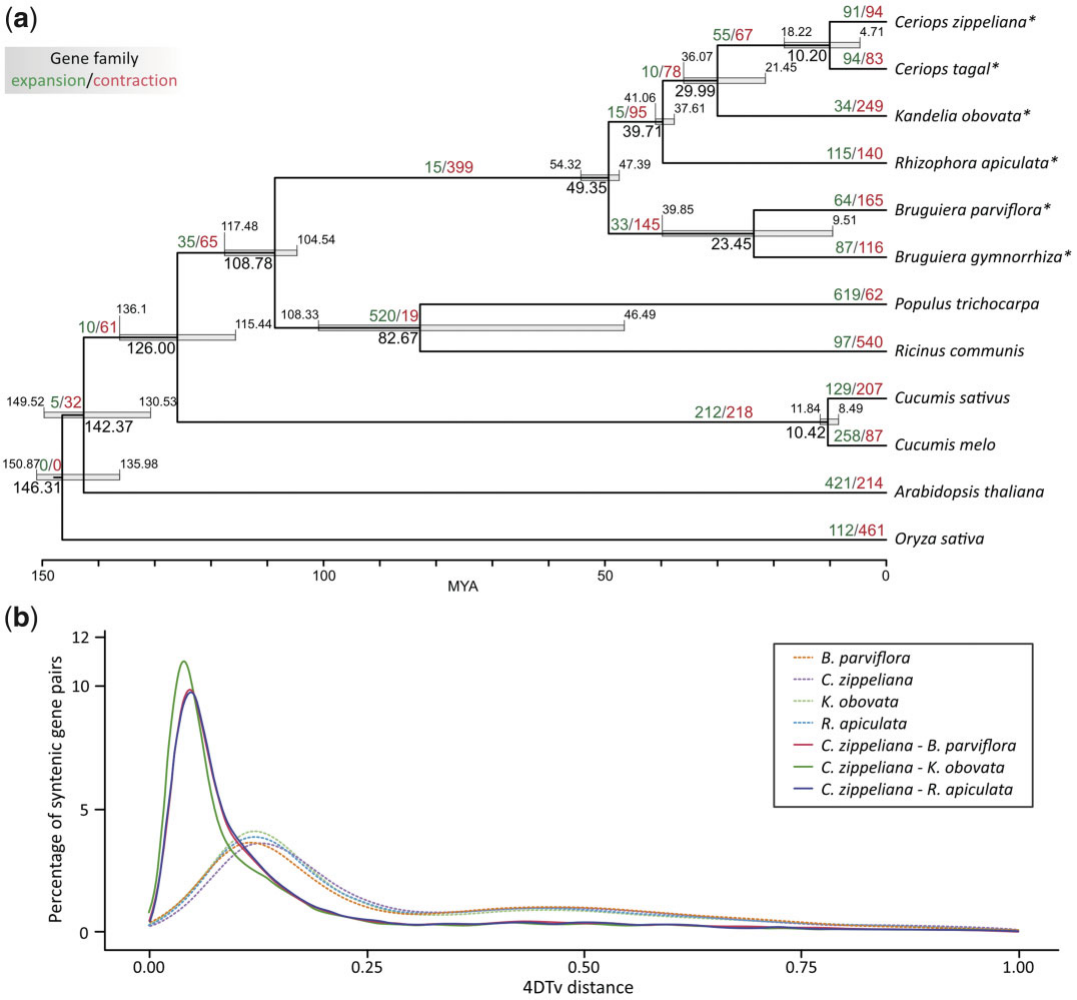
Comparative analysis and phylogenetic tree of *C. zippeliana* and other plant species. a) A maximum-likelihood phylogenetic tree of six *Rhizophoreae mangroves* (indicated with asterisks) and other plant species based on single-copy orthologous protein sequences. Numbers at each node represent the estimated divergence time in MYA with gray bars showing the corresponding 95% equal-tail credibility intervals. The number of genes in expanded and contracted families is indicated in green and red, respectively. b) Distribution of 4DTv distances between orthologous genes (solid line) and paralogous genes (dotted line) in *C. zippeliana, B. parviflora*, *K. obovata*, and *R. apiculata.*

To explore the relationships among mangrove and other plant species, gene sets from six Rhizophoraceae (*B. parviflora*, *B. gymnorhiza*, *C. tagal*, *C. zippeliana*, *K. obovata*, and *R. apiculata*), five dicots (*A. thaliana*, *C. melo*, *C. sativus*, *P. trichocarpa*, and *R. communis*) and one monocot (*O. sativa*) were analyzed. A total of 332,172 proteins (out of 348,758 input proteins from 12 species; 95.24%) were clustered into 23,233 orthologous groups. We constructed a maximum-likelihood tree based on sequence information from single-copy orthologs and estimated the divergence time based on the topology and branch lengths. *C.* *zippeliana* and *C. tagal* diverged approximately 10.2 (4.7–18.2) MYA (Fig. 4a), and their last common ancestor diverged from *K. obovata* 29.9 (21.4–36.1) MYA. The last common ancestor of *B. parviflora* and *B. gymnorhiza* diverged from the last common ancestor of *Ceriops, Kandelia*, and *Rhizophora* roughly 49.3 (47.3–54.3) MYA (Fig. 4a). The analysis of age distributions of transversion substitutions at 4-fold degenerate synonymous sites (4DTv) based on 5,997 pairs of paralogous genes residing within 508 syntenic blocks clearly indicated that *C. zippeliana* has undergone a whole-genome duplication event. This result was supported by the extensive presence of intragenomic orthologous syntenic regions throughout the genome (Fig. 1). There was a one-to-one relationship between most chromosome pairs, with a more complex relationship among chromosomes 3, 7, and 11. Interestingly, we observed a common whole-genome duplication event occurring during the same period in *B. parviflora, K. obovata*, and *R. apiculata* (Fig. 4b), and this shared genome-wide duplication appeared to predate the divergence of the Rhizophoraceae members. Examination of synteny between *C. zippeliana* and closely related mangroves also revealed an extensive degree of macrosynteny conservation in among Rhizophoraceae species investigated (*B. parviflora*, *K. obovata*, and *R. apiculata*; Supplementary Fig. 1).

## Data availability

The *C. zippeliana* genome assembly was deposited in the DDBJ/ENA/GenBank database under the accession number JAHLKL000000000. RNA-seq data (MGISEQ) were submitted to the NCBI Sequence Read Archive (SRA) database under BioProject accession number PRJNA734101 (SRR16555174).

Supplemental material is available at *G3* online.

## Supplementary Material

jkac025_Supplementary_Data
