# Supplementary material for: A de novo reference assembly of the yellow mangrove Ceriops zippeliana genome
Source: G3 (Bethesda). 2022 Feb 2;12(4):jkac025. doi: 10.1093/g3journal/jkac025 (PMC8982413; doi:10.1093/g3journal/jkac025)
Supplement: jkac025_Supplementary_Data [file jkac025_supplementary_data.pdf]

(A)

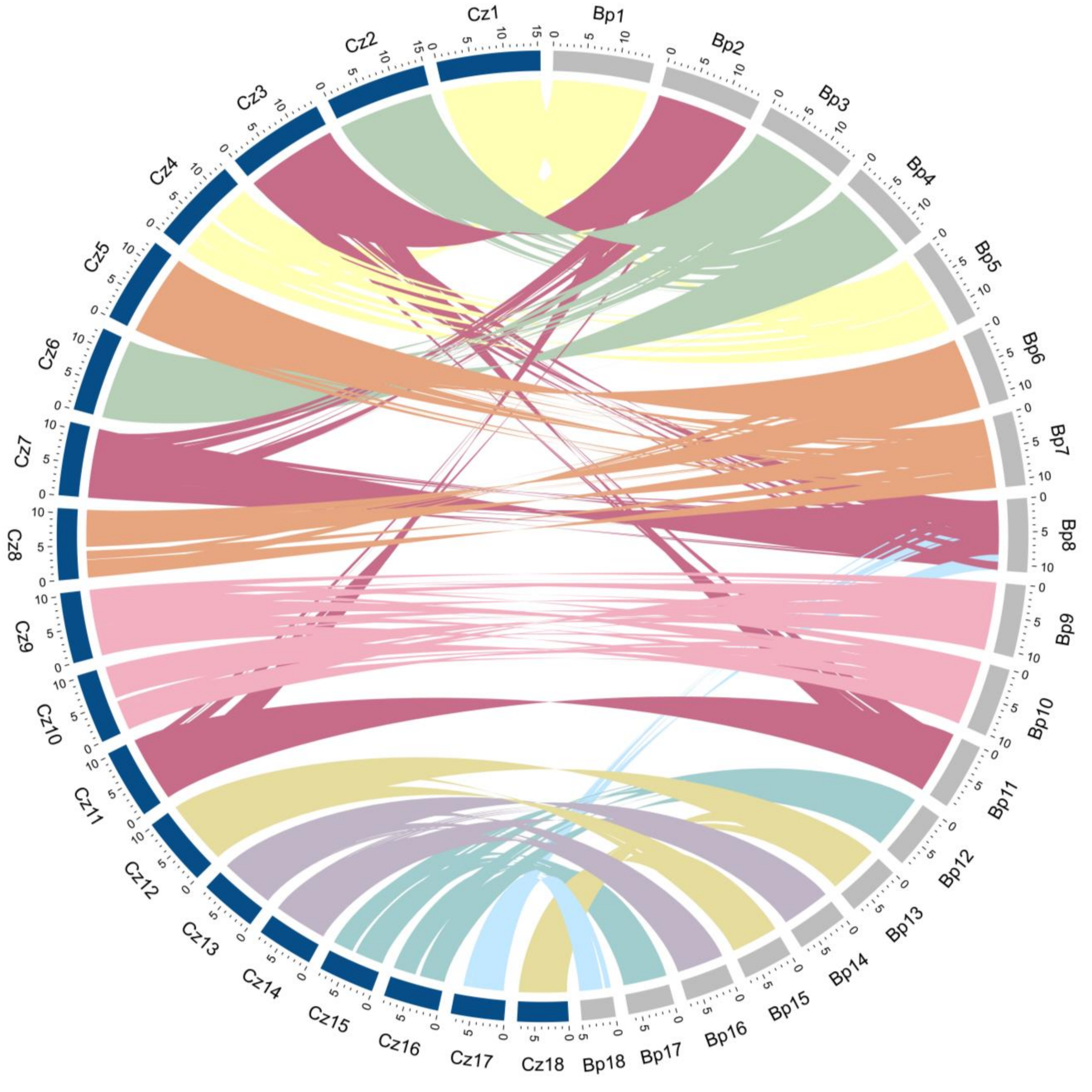
Cz1
Cz2
Cz3
Cz4
Cz5
Cz6
Cz7
Cz8
Cz9
Cz10
Cz11
Cz12
Cz13
Cz14
Cz15
Cz16
Cz17
Cz18
Bp1
Bp2
Bp3
Bp4
Bp5
Bp6
Bp7
Bp8
Bp9
Bp10
Bp11
Bp12
Bp13
Bp14
Bp15
Bp16
Bp17
Bp18

(B)

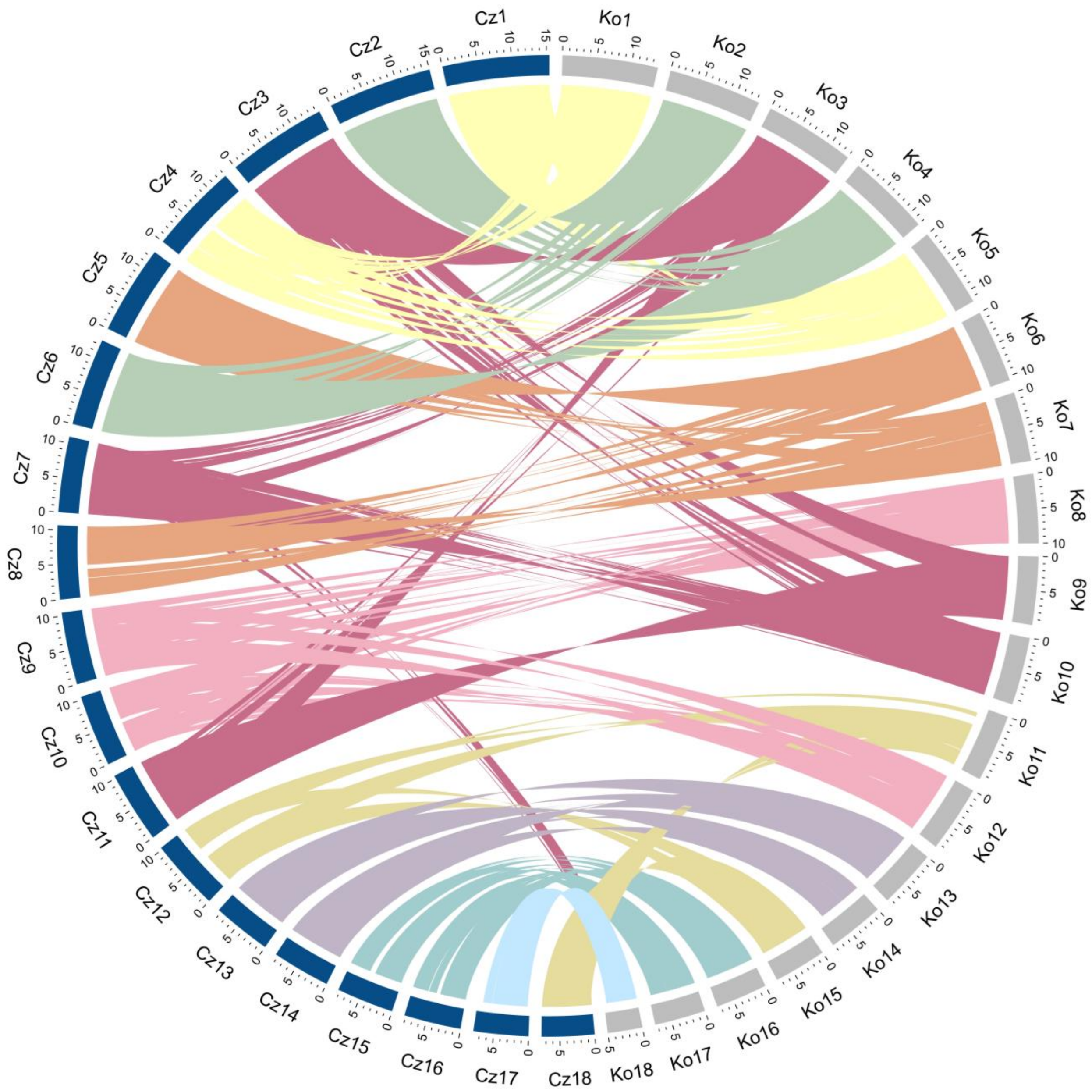
Cz1
Cz2
Cz3
Cz4
Cz5
Cz6
Cz7
Cz8
Cz9
Cz10
Cz11
Cz12
Cz13
Cz14
Cz15
Cz16
Cz17
Cz18
Ko1
Ko2
Ko3
Ko4
Ko5
Ko6
Ko7
Ko8
Ko9
Ko10
Ko11
Ko12
Ko13
Ko14
Ko15
Ko16
Ko17
Ko18

(C)

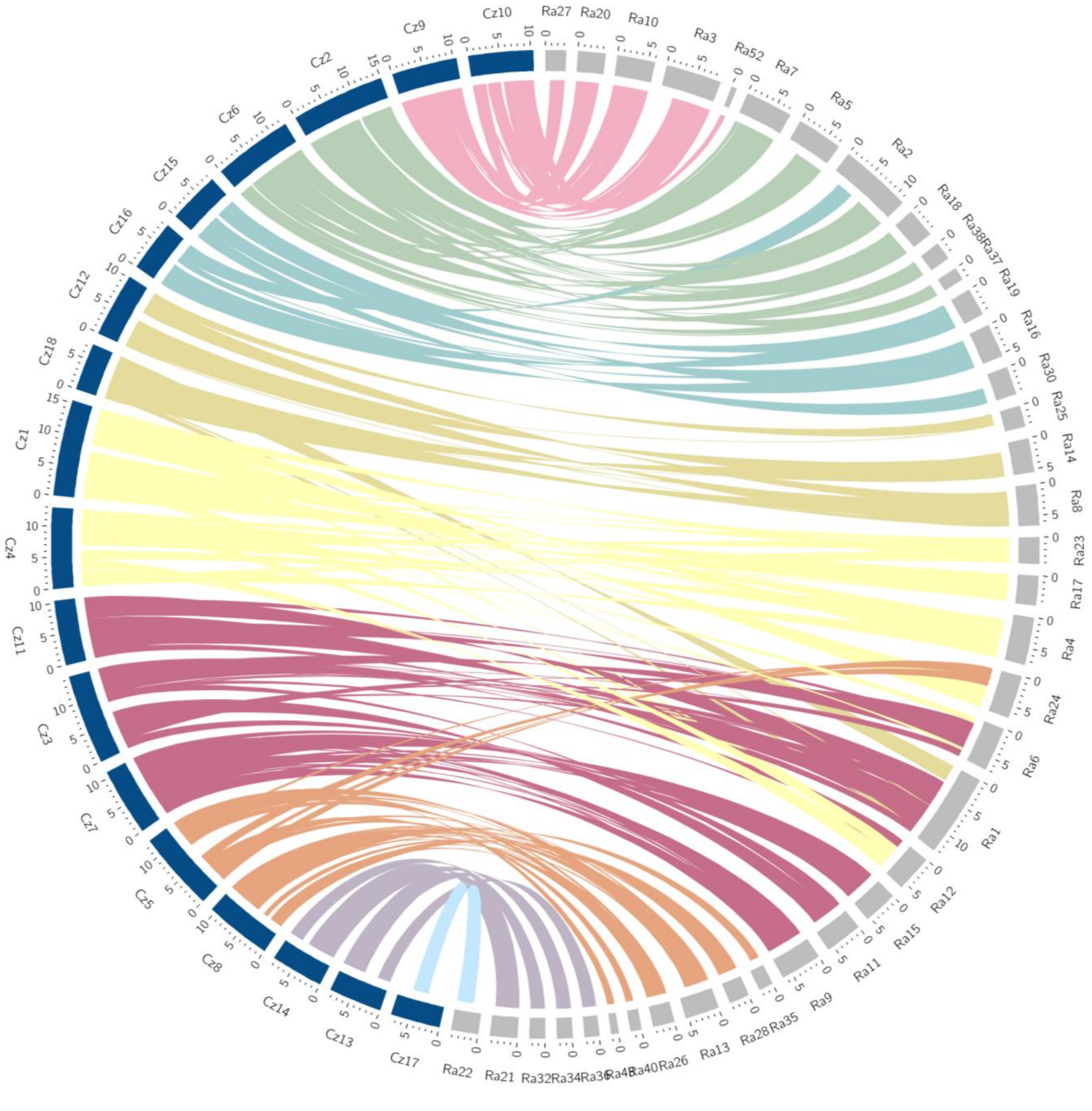

Supplementary Figure 1. Synteny between *C. zippeliana* and other closely related mangrove species: (A) *B. parviflora* (B) *K. obovata* (C) *R. apiculata*).
